# COVID-19 Vaccine-Induced Cerebral Sinus Thrombosis: Coincidence vs. Cause?

**DOI:** 10.7759/cureus.26436

**Published:** 2022-06-29

**Authors:** Abdalla Fadul, ELMustafa Abdalla, Elabbass Abdelmahmuod, Mohammed Abdulgayoom, Elrazi Ali, Akram Al-warqi, Hani Al-yahary

**Affiliations:** 1 Internal Medicine Department, Hamad Medical Corporation, Doha, QAT; 2 Internal Medicine Department, Hamad General Hospital, Doha, QAT; 3 Radiology Department, Hamad Medical Corporation, Doha, QAT

**Keywords:** thrombosis, cerebral venous sinus thrombosis (cvst), moderna vaccine, covid-19 vaccine, covid 19

## Abstract

Patients who were vaccinated against COVID-19 have experienced thrombosis-thrombocytopenia syndrome and cerebral venous sinus thrombosis (CVST). It is important to be aware of this potential side effect of the vaccine and to be able to recognize early clinical symptoms and signs of CVST.

In this paper, we present two cases of COVID-19 vaccination-related CVST. The patients who suffered headaches and seizures were found to have CVST, which was treated with anticoagulation.

## Introduction

Cerebral venous sinus thrombosis (CVST) is less prevalent than most other causes of stroke (It accounts for 0.5%-1% of all stroke causes). Still, it is more challenging to identify, as it has been found to have a broader clinical scope than previously thought [1]. Hereditary prothrombotic disorders, antiphospholipid antibody syndrome, cancer, pregnancy, autoimmune illnesses, and infections are all common causes of CVST [2]. On another note, at the end of 2019, a new coronavirus strain was identified as the source of a cluster of pneumonia cases in Wuhan, Hubei Province, China. It quickly spread worldwide, culminating in a global pandemic that is now known as the COVID-19 pandemic [3]. The most promising strategy for containing the COVID-19 pandemic is the development of vaccines to prevent SARS-CoV-2 infection. At present, COVID-19 vaccinations are widely accessible across the world [4]. The most common side effects of COVID-19 immunization are a local response at the injection site, followed by non-specific upper respiratory tract symptoms. However, there are also rare instances of vaccine-induced CVST. Here we present and study two cases of it [5].

## Case presentation

Case 1

**Figure 1:**
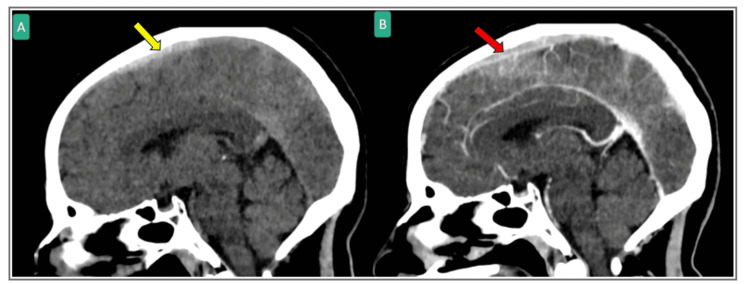
Sagittal head CT scan with non-contrast (A) and with contrast (B) showing hyperdensity at the superior sagittal sinus (Yellow arrow) that was confirmed by contrast to be superior sagittal sinus contrast filling defect (red arrow) suggesting cerebral venous thrombosis.

**Figure 2:**
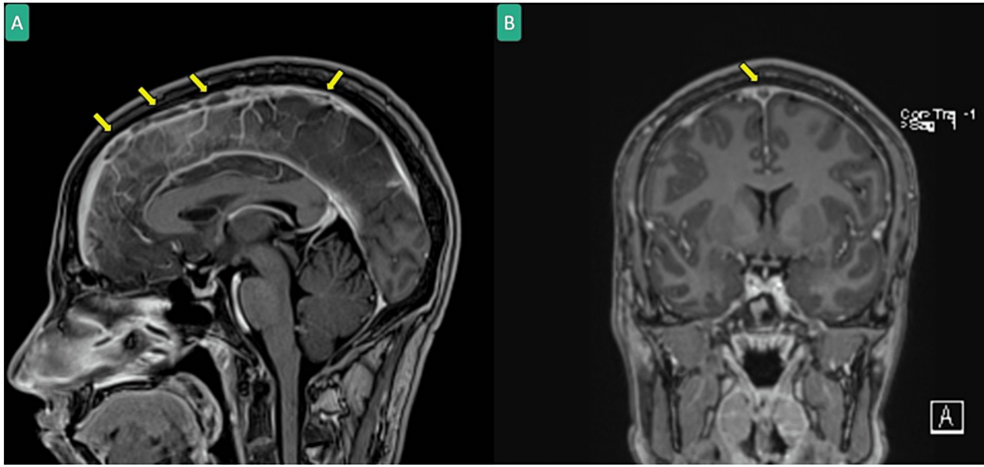
Head MRI with contrast in MPRAGE sequence sagittal (A) and coronal (B) showing extensive superior sagittal sinus thrombosis (yellow arrows). MRI: Magnetic resonance imaging

A 29-year-old gentleman with no past medical history was presented to the emergency department with complaints of generalized severe thunderclap headache associated with vomiting for five days. His symptoms started ten days after he received the third dose of the Moderna COVID vaccine. No adverse reactions were reported after he had received the first two doses of the vaccine. His physical examinations were unremarkable. His basic labs were within the normal range. However, the CT venogram intracranial showed the presence of extensive venous sinus thrombosis (see Figure 1), which was confirmed by cranial magnetic resonance venography (MRV) (see Figure 2).

Further investigations were ordered, including thrombophilia and coagulation profiles, all of which were negative (see Table 1 and Table 2, respectively). His viral screening, including screening for COVID-19, was negative. The neurology team started him on a course of enoxaparin 80 mg BID, then shifted to dabigatran 150 mg BID, and later discharged him with a recommendation of neurology outpatient follow-up.

**Table 1:** Complete blood count (CBC) and coagulation profile for Case 1 WBC: White blood count; RBC: Red blood cell; Hgb: Hemoglobin; Hct: Hematocrit; MCV: Mean corpuscular volume; MCH: Mean corpuscular hemoglobin; MCHC: Mean corpuscular hemoglobin concentration; RDW-CV: Red cell distribution width - coefficient of variation; INR: International normalized ratio; APTT: Activated partial thromboplastin time.

	Value (Unit)	Normal Range
WBC	3.0 x 10^3^/uL	4.0-10.0
RBC	5.2 x 10^6^/uL	4.5-5.5
Hgb	15.9 gm/dL	13.0-17.0
Hct	47.2%	40.0-50.0
MCV	90.2 fL	83.0-101.0
MCH	30.4 pg	27.0-32.0
MCHC	33.7 gm/dL	31.5-34.5
RDW-CV	12.2%	11.6-14.5
Platelet	99 x 10^3^/uL	150-400
Prothrombin Time	12.7 seconds	9.7-11.8
INR	1.2	0.8-1.1
APTT	33.6 seconds	24.6-31.2

**Table 2:** Thrombophilia workup for Case 1 ANCA: Antineutrophil cytoplasmic antibodies; Ab: Antibody

	Value (Units)	Normal Range
Lupus Screen	40.1 seconds	30.4-45.3
Protein C Activity	114.5%	70.0-140.0
Protein S Activity	>129.0%	72.0-126.0
ANCA	Negative	
Anti Cardiolipin Ab IgG	1.00 GPL	<10.0 GPL
Anti Cardiolipin Ab IgM	<0.80 MPL	0-12 MPL

Case 2

**Figure 3:**
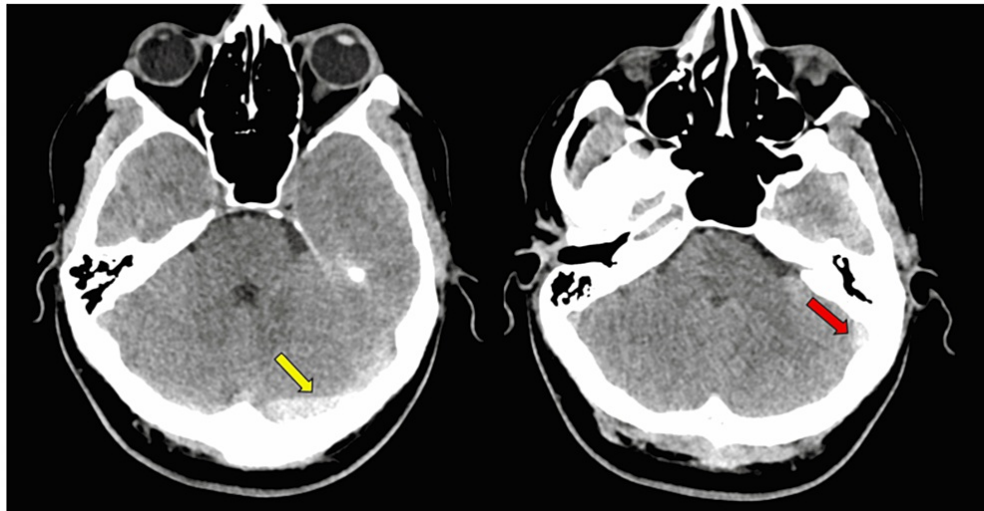
Axial head CT scan showing hyperdensity suggesting cerebral venous thrombosis within the left transverse sinus (yellow arrow) and beginning of the sigmoid sinus (red arrow).

**Figure 4:**
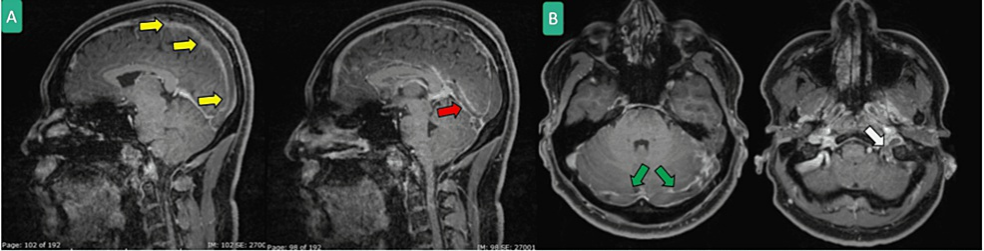
Head MRI with contrast MPRAGE sequence sagittal (A) and axial (B) showing extensive cerebral venous thrombosis in superior sagittal (yellow arrows), straight (red arrow), bilateral transverse (green arrow) and sigmoid/jugular sinuses (white arrows).

A 31-year-old gentleman with no past medical history was presented to the emergency department with recurrent generalized tonic-clonic seizures and five episodes associated with tongue bites, which lasted for a few minutes, followed by regained consciousness. After recovery, he complained of a headache. The patient had taken the Moderna vaccine three weeks before his presentation. His physical exams were unremarkable. An urgent head CT showed the presence of hyperdense mid-superior sagittal sinus with filling defects on post-contrast images, suggestive of superior sagittal sinus thrombosis (see Figure 3). An MRV was done, which showed the redemonstration of superior sagittal sinus thrombosis with no interval progression and no evidence of related brain insult (see Figure 4). The patient was diagnosed with cerebral venous thrombosis, started on enoxaparin 60 mg BID and discharged on warfarin 6 mg and levetiracetam 500 BID with a recommendation of neurology follow-up.

## Discussion

Several vaccination emergency usage lists (EUL) have been published by the World Health Organization (WHO). They mention several vaccinations, including the Comirnaty vaccine from Pfizer and BioNTech, the SII/Covishield and AstraZeneca/AZD1222 vaccine, the Janssen/Ad26.COV 2.S vaccine, and the Moderna COVID-19 vaccine (mRNA 1273) [6]. While immunization has been proven safe and effective, the constant increase in adverse reactions has damaged public faith in the immunization program [7]. While these adverse effects are uncommon, they can result in severe morbidity and mortality and should be taken into consideration. Vaccine-induced immune thrombocytopenia and thrombosis (VITT) were the most dangerous and fatal, especially in young people and women [8]. Even though the actual incidence of VITT is still unknown, reports have detailed only a tiny number of cases among the tens of millions of people who have received vaccinations. Although VITT seldom occurs, it is associated with a significant mortality rate [9].

The underlying pathophysiology could be related to antibody-mediated thrombotic thrombocytopenia during COVID-19, which is thought to be an autoimmune response triggered by SARS-CoV-2. Due to the increased risk of thrombotic thromboembolic events associated with severe COVID-19, affected individuals are frequently given heparin. An undiagnosed COVID-19 infection could explain these findings in vaccination recipients [10].

VITT is related to elevated levels of antibodies against platelet factor 4 (PF4), a protein that stimulates platelets to form new blood vessels. It is a rare syndrome similar to heparin-induced thrombocytopenia, where the same antibodies are detected following heparin exposure [11]. According to some researchers, patients who report thrombosis and average platelet counts following vaccination may be in the early stages of VITT. These patients should be continuously assessed for the development of thrombocytopenia [12].

Fondaparinux, argatroban, and direct oral anticoagulants (e.g., apixaban or rivaroxaban) should be considered the treatment of choice when the platelet count of the patient is above 50,000 and there is no significant bleeding risk. Within a few days of starting anticoagulation treatment, intravenous immunoglobulins and glucocorticoids may increase the platelet count and minimize the risk of hemorrhagic transformation [13]. Anticoagulants and antiplatelet medications are not recommended for routine thromboprophylaxis to prevent thrombosis following the administration of the Moderna COVID-19 vaccine [14].

## Conclusions

Clinicians must consider the possibility of cerebral sinus thrombosis in patients who report post-vaccination seizures or headaches. Surprisingly, most cases are documented in younger individuals, as is the situation with our patients. We hope this paper will raise awareness of the condition and encourage more patients to come forward for treatment, as many cases may have gone unrecognized and unreported.

## References

[1] Abdulgayoom M, Abdelmahmuod E, Elfaki A, Halabiya MA (2021). Cerebral venous sinus thrombosis as an unexpected complication of COVID-19 pneumonia. Cureus.

[2] Qader AQ, Nahzat O, Qader AJ, Atahi S (2021). A case report of cerebral venous infarction due to venous sinus thrombosis as complication in a Covid-19 patient. Radiol Case Rep.

[3] Ali E, Badawi M, Abdelmahmuod E, Kohla S, Yassin MA (2020). Chronic lymphocytic leukemia concomitant with COVID 19: a case report. Am J Case Rep.

[4] Abdulgayoom M, Albuni MK, Abdelmahmuod E, Murshed K, Eldeeb Y (2021). Minimal change nephrotic syndrome four days after the administration of Pfizer-BioNTech COVID-19 vaccine-a new side effect or coincidence?. Clin Case Rep.

[5] Alamin MA, Abdulgayoom M, Niraula S, Abdelmahmuod E, Ahmed AO, Danjuma MI (2021). Rhino-orbital Mucormycosis as a complication of severe COVID-19 pneumonia. IDCases.

[6] Dakay K, Cooper J, Bloomfield J (2021). Cerebral venous sinus thrombosis in COVID-19 infection: a case series and review of the literature. J Stroke Cerebrovasc Dis.

[7] Meo SA, Bukhari IA, Akram J, Meo AS, Klonoff DC (2021). COVID-19 vaccines: comparison of biological, pharmacological characteristics and adverse effects of Pfizer/BioNTech and Moderna vaccines. Eur Rev Med Pharmacol Sci.

[8] Guendouz C, Quenardelle V, Riou-Comte N (2021). Pathogeny of cerebral venous thrombosis in SARS-Cov-2 infection: case reports. Medicine (Baltimore).

[9] Lima Miranda O, Pereira A, Castro M (2021). Paraganglioma: an unexpected diagnosis in a patient with cerebral venous sinus thrombosis and SARS-CoV-2 infection. Cureus.

[10] Huang Z, Su Y, Zhang T, Xia N (2022). A review of the safety and efficacy of current COVID-19 vaccines. Front Med.

[11] Shiri T, Evans M, Talarico CA (2022). The population-wide risk-benefit profile of extending the primary COVID-19 vaccine course compared with an mRNA booster dose program. Vaccines (Basel).

[12] See I, Su JR, Lale A (2021). US case reports of cerebral venous sinus thrombosis with thrombocytopenia after Ad26.COV2.S vaccination, March 2 to April 21, 2021. JAMA.

[13] Waqar U, Ahmed S, Gardezi SM (2021). Thrombosis with thrombocytopenia syndrome after administration of AZD1222 or Ad26.COV2.S vaccine for COVID-19: a systematic review. Clin Appl Thromb Hemost.

[14] Long B, Bridwell R, Gottlieb M (2021). Thrombosis with thrombocytopenia syndrome associated with COVID-19 vaccines. Am J Emerg Med.

